# P-1747. Cost Avoidance Analysis of Outpatient Parenteral Antimicrobial Therapy (OPAT) Plan Reconciliation on Patients Enrolled in Value-Based Payment Models at an Academic Hospital

**DOI:** 10.1093/ofid/ofae631.1910

**Published:** 2025-01-29

**Authors:** Jennifer Ross, Kimberly Boeser, Matt Hordyk, Bhupinder Manhani, Elizabeth B Hirsch, Michael Evans, William D Sieling, Susan E Kline, Alison Galdys

**Affiliations:** M Health Fairview - University of Minnesota Medical Center, Minneapolis, Minnesota; University of Minnesota Medical Center, Minneapolis, MN; Fairview Health Services, Coon Rapids, Minnesota; Fairview Health Services, Coon Rapids, Minnesota; University of Minnesota College of Pharmacy, Minneapolis, MN; University of Minnesota, Minneapolis, Minnesota; University of Minnesota Medical School, Minneapolis, MN; University of Minnesota Medical School, Minneapolis, MN; University of Minnesota, Minneapolis, Minnesota

## Abstract

**Background:**

Outpatient parenteral antimicrobial therapy (OPAT) has many potential benefits, including avoidance of hospitalizations, reduced length of stay, and prevention of hospital-associated conditions. However, suboptimal OPAT delivery can result in readmissions and emergency department (ED) visits—outcomes that are unfavorable for patients and disincentivized within value-based care (VBC) models. Structured OPAT programs have been shown to decrease unscheduled healthcare use. Total cost of care (TCOC) analyses for OPAT program justification have not been well described. We therefore analyzed TCOC for a cohort of OPAT recipients enrolled in VBC pre- and post-implementation of OPAT plan reconciliation in June 2020.

**Methods:**

Unique, adult OPAT patients discharged to home or post-acute care facilities from an academic hospital between 6/2019 and 6/2022 were included. Infectious diseases (ID) pharmacist reconciliation of OPAT plans prior to hospital discharge launched on 6/14/2020; all OPAT recipients discharged on or after this date were included in the post-implementation cohort. Average one-year post-intervention TCOC avoidance per patient was calculated using patient charges, a proxy for payor cost. The percentage of patients enrolled in a VBC plan was determined to calculate implied TCOC reduction annually.

**Results:**

Nine hundred sixty-two OPAT patients were included: 198 pre- and 764 post-implementation. Fewer patients were included in the pre-intervention group given unretrievable financial data due to archiving rules. In our cohort, 16.9% of patients were enrolled in a VBC plan. Both pre- and post-intervention cohorts showed an increase in TCOC after index OPAT hospitalization; pre-intervention at 155% and post at 29%. This yielded a post-intervention average one-year cost avoidance of $15,754.55/patient. Based on our institution’s historical OPAT volume of 950 patients cared for annually, these data equate to an implied TCOC reduction of $2,529,409/year.

**Conclusion:**

Implementation of formal ID pharmacist reconciliation of OPAT plans generated significant cost avoidance amongst OPAT patients enrolled in VBC plans. This is evidence that outcomes incentivized by VBC models align with outcomes facilitated by structured OPAT programs.

**Disclosures:**

**Jennifer Ross, PharmD, BCIDP**, Shionogi Inc.: Advisor/Consultant **Elizabeth B. Hirsch, PharmD, FCCP, FIDSA**, GSK: Advisor/Consultant|GSK: Honoraria

